# An outbreak of feline infectious peritonitis in a Taiwanese shelter: epidemiologic and molecular evidence for horizontal transmission of a novel type II feline coronavirus

**DOI:** 10.1186/1297-9716-44-57

**Published:** 2013-07-17

**Authors:** Ying-Ting Wang, Bi-Ling Su, Li-En Hsieh, Ling-Ling Chueh

**Affiliations:** 1 Graduate Institute of Veterinary Medicine, School of Veterinary Medicine, National Taiwan University, Taipei 10617, Taiwan; 2 Graduate Institute of Veterinary Clinical Sciences, School of Veterinary Medicine, National Taiwan University, Taipei 10617, Taiwan

## Abstract

Feline infectious peritonitis (FIP) is a fatal disease caused by feline coronavirus (FCoV) infection. FCoV can be divided into serotypes I and II. The virus that causes FIP (FIPV) is believed to occur sporadically and spread infrequently from cat to cat. Recently, an FIP outbreak from an animal shelter was confirmed in Taiwan. FCoV from all the cats in this shelter were analyzed to determine the epidemiology of this outbreak. Thirteen of 46 (28.2%) cats with typical signs of FIP were identified. Among them, seven cats were confirmed by necropsy and/or histopathological examinations. Despite the fact that more than one FCoV was identified in this multi-cat environment, the eight FIP cats were invariably found to be infected with a type II FCoV. Sequence analysis revealed that the type II FIPV detected from fecal samples, body effusions and granulomatous tissue homogenates from the cats that succumbed to FIP all harbored an identical recombination site in their *S* gene. Two of the cats that succumbed to FIP were found to harbor an identical nonsense mutation in the *3c* gene. Fecal shedding of this type II virus in the effusive form of FIP can be detected up to six days before death. Taken together, our data demonstrate that horizontal transmission of FIPV is possible and that FIP cats can pose a potential risk to other cats living in the same environment.

## Introduction

Feline infectious peritonitis (FIP) is a fatal disease in cats caused by feline coronavirus (FCoV). FCoV is an enveloped, positive-stranded RNA virus belonging to genus *Alphacoronavirus*, family *Coronaviridae*, within the order *Nidovirales*. The genome size of FCoV is approximately 28.9 kb, including a non-structural replicase gene; four structural genes encoding the spike (S), envelope, membrane and nucleocapsid proteins; and five accessory genes *3ab*c and *7ab*[1].

Feline coronaviruses cause mild to inapparent and transient infections of the gut and are ubiquitous in cat populations worldwide [2]. They exist in two serotypes, I and II [3]. Type I FCoV is predominant in the field, whereas type II virus represents only 2-30% of infection [4-8]. Accumulating genetic evidence indicates that type II FCoV have arisen by two homologous recombinations between type I FCoV and canine CoV (CCoV) [9,10]. Both serotypes can mutate in the host to acquire macrophage tropism and cause a systemic disease known as feline infectious peritonitis [2,11,12]. Due to a lack of virus-shedding in studies of FIP cats, the mutant FIP viruses (FIP causing FCoV, FIPV) are presumably contained only within the diseased tissues and not transmitted by cat-to-cat contact under natural circumstances [2,11,13,14].

In this paper, we report herein on an epizootic of FIP in a Taiwanese shelter that was caused by a novel type II FCoV. Epidemiological and molecular studies of isolates from various healthy and affected cats in this shelter strongly suggest that this virus was brought in by the introduction of kittens from another shelter with subsequent horizontal spread to co-housed adult cats.

## Materials and methods

### Animals and specimen collection

A total of 46 cats from a private cat shelter were subjected to this study from September 2011 to August 2012. The shelter houses adult cats and occasionally some kittens. All of the cats were either stray animals or had been rescued, and some of them were being temporally raised at the homes of various private cat rescuers. Before this outbreak, all cats were living together in a cageless indoor environment and shared food, water bowls and litter boxes. Some of the cats were siblings, and the others were unrelated (Table 1).

**Table 1:** Information for all FIP-suspected and confirmed cats from the cat shelter

**Cat no**	**Age**^**1**^	**Date of enter the shelter**	**Date of fever onset**	**Date of death**	**Clinical findings**	**Necropsy findings**	**Effusive/non-effusive**
1	3m	Jun. 16, 2011	Aug. 17, 2011	Sep. 01, 2011	Fever, anorexia, ascites, neurological signs		
2^a^	4m	Aug. 06, 2011	NA^2^	Sep. 21, 2011	Clinical signs unavailable		
3^b^	3m	Jul. 11, 2011	Aug. 18, 2011	Sep. 25, 2011	Fever, anorexia, weight loss, neurological signs		
4	2.5m	Jun. 08, 2011	Aug. 16, 2011	Sep. 28, 2011	Fever, ascites, neurological signs		
5^a^	4m	Aug. 06, 2011	Aug. 15, 2011	Oct. 20, 2011	Fever, pleural effusion, diarrhea		
6	7m	Apr. 24, 2011	NA	Oct. 22, 2011	Anorexia, weight loss, neurological signs		
7	3y6m	Resident	NA	Oct. 27, 2011		Ascites, jaundice, granulomatous lesion in kidney, fibrinous peritonitis	Effusive
8	6m	Jul. 11, 2011	NA	Dec. 14, 2011		Granulomatous changes in kidney, liver, lung, brain and eyes	Non-effusive
9	2y	Resident	NA	Dec. 28, 2011		Ascites, pleural effusion and pericardial effusion, granulomatous changes in kidney, liver and intestine	Effusive/non-effusive
10^b^	3m	Jul. 11, 2011	NA	Nov. 05, 2011		Granulomatous changes in kidney, liver and omentum	Non-effusive
11^c^	1y6m	Resident	NA	Feb. 14, 2012		Ascites and pleural effusion, jaundice, fibrinous peritonitis, granulomatous changes in kidney, liver, lung and spleen.	Effusive/non-effusive
12^c^	1y6m	Resident	NA	Mar. 19, 2012		Jaundice, fibrinous peritonitis, granulomatous changes in thoracic and abdominal wall, kidney, liver, lung, spleen omentum, and eyes.	Effusive/non-effusive
13	1y7m	Resident	NA	Apr. 13, 2012		Jaundice, enlargement of liver and mesenteric lymph node, granulomatous changes in kidney and lung.	Non-effusive

^1^ Age of the cats when clinical signs of FIP appeared.

^2^ Not available.

^a, b, c^: Siblings.

Feces or rectal swab samples of all asymptomatic cats were collected at least once to monitor the presence of FCoV. For cats showing signs of illness suspected to be FIP, body effusions, whole blood and swab samples, including rectal, nasal, oral and conjuctival swabs, were collected routinely. In addition to supportive care, these FIP-suspected animals were subjected to treatment with prednisolone (Prelon®, YF Chemical Corp., New Taipei City, Taiwan), benazepril (Cibacen®, Novartis, Barbera del Valles, Spain) and recombinant human interferon alpha (Roferon®-A, Roche, Basel, Switzerland). Cats that succumbed to illness were subjected to necropsy for pathological confirmation. Upon necropsy, body effusions were first drawn by needle and syringe, followed by collecting swabs, whole blood, urine and granulomatous lesions in the internal organs. All samples were frozen at -20 °C until use. All specimens were screened for FCoV by reverse transcription-nested polymerase chain reaction (RT-nPCR) [15]. The specimens that tested positively were subsequently subjected to further analysis.

### Sample preparation and reverse transcription

Swab samples were suspended in 1 mL of 0.1% diethyl pyrocarbonate (DEPC)-treated water. Fecal samples were suspended with 9 times of 0.1% DEPC-treated water by vortexing. The suspension was centrifuged, and the supernatant was transferred into a new tube. Approximately 0.5 g tissues were frozen and then ground with a mortar and pestle in the presence of 2 mL of Trizol [16]. Total RNA was extracted from 300 μL of swab suspension, whole blood, fecal suspension, tissue homogenate and body effusion using Trizol. Twenty-one microliters of isolated RNA were reverse transcribed with template-specific primer N1 (5′-gctacaattgtatcctcaac-3′) or P211 [15] with Moloney murine leukemia virus reverse transcriptase (Invitrogen, CA, USA). The reaction was incubated at 37°C for 60 min, 72°C for 15 min and finally, at 94°C for 5 min.

### Typing of FCoV using nested PCR

For typing of FCoV, nested PCR was performed according to the procedures reported by Addie et al. [5] with slight modification. Following reverse transcription, 5 μL of the complementary DNA was added to 25 μL of the PCR mixture (Invitrogen, CA, USA) according to the manufacturer’s instructions for the following primer sets: S1 and Iffs for type I FCoV detection and S1 and Icfs for type II FCoV detection. Nested PCR was performed on 2 μL of the first PCR product using nested primers. The expected size of the second PCR yielded for type I and II FCoV were 360 and 218 bp, respectively. The products of RT-nPCR were electrophoresed, and then, target DNA fragments were purified (Geneaid Biotech, Ltd, Taipei) and sequenced (Mission Biotech, Taipei, Taiwan) from both orientations.

### Amplification, sequencing and analysis of *3a* and *3c* gene from the type II FCoV

To amplify the *3a* gene of type II FCoV from FIP cats, a specific primer set that can amplify from the type II *S* gene to the *3a* gene was designed. Complementary DNA amplified with the primer set targeted the 3′ end of the type II FCoV *S* gene (Icfs) and 5′ end of the FCoV *3a* gene (3aR2: 5′-caccaaaacctatacacacaag-3′). The temperature cycling consisted of 5 min of preheating at 94°C; 35 cycles of denaturation at 94°C for 20 s, annealing at 50°C for 20 s and extension at 72°C for 30 s; and a final extension at 72°C for 5 min. Following a second round of amplification with the primers nIcfs and 3aR2, the expected size of the product was approximately 600 bp. The amplicons were electrophoresed, purified and sequenced from both orientations to confirm the nucleotide sequences.

To amplify the *3c* gene of type II FCoV from FIP cats, specific primer sets that can amplify from the 3′ end of the type II *S* gene to the *3c* gene were designed. Complementary DNA were amplified with the forward primer (Icfs) and reverse primer (E68R: 5′-aatatcaatataattatctgctgga-3′ and/or N21R: 5′-gttcatctccccagttgacg-3′). The temperature cycling consisted of 5 min of preheating at 94°C; 40 cycles of denaturation at 94°C for 30 s, annealing at 46°C for 30 s and extension at 72°C for 90 s; and a final extension at 72°C for 7 min. Following a second round of amplification with primer nIcfs and E68R, the products were electrophoresed, purified and sequenced from both orientations to confirm the nucleotide sequences.

### Phylogenetic analysis and recombination site analysis of a type II FCoV

Multiple sequence alignments were preformed with ClustalW 2.0 and manually edited in EditSeq (DNASTAR, Madison, USA). Phylogenetic analyses were conducted using MegAlign, version 7.2.1 (DNASTAR, Madison, USA). Bootscan and similarity graphs were prepared with SimPlot 3.5.1 software (SCRoftware, Baltimore, USA).

## Results

### Confirmation of an FIP outbreak in the cat shelter

The shelter had been ongoing for three-and–a-half years. No history of FIP was recorded before August 2011. Kittens (cat 1, 3, 4, 8 and 10) were introduced to this shelter between June and July 2011. After entering, these kittens played together and lived with the resident adult cats. Before the outbreak, the kittens were taken individually to a veterinarian for vaccinations and attended adoption activities. Fever was first observed in four of the kittens (cat 1, 3, 4, 5) within a period of a few days (from August 15_th_ to 18_th_) (Table 1). Clinical signs, e.g., fever, anorexia, neurological signs, panting and/or abdominal extension were observed for the following two months and the kittens died sequentially between September 1_st_ and October 22_nd_ (Table 1). The caregivers at the shelter requested our help on September 27_th_. All of the resident cats in the shelter were immediately screened for FCoV using RT-nPCR. All FCoV positive cats were isolated individually and kept separated. Nevertheless, starting in September, some of the adult cats with FIP (cats 7-13) showed clinical signs similar to those of the kittens and all of them died later.

Six kittens (cats 1-6) with body effusions and/or neurological signs succumbed within the first two months without necropsy confirmation (Table 1). Cat 1 was once presented to our teaching hospital, and ascites was taken from the cat. In cats 7-13, typical necropsy features, characterized by ascites and/or pleural effusions in the body cavities (effusive FIP) and granulomatous lesions in several organs, especially the kidney, liver, lung, omentum and eyes (non-effusive FIP), were found. Cats 9, 11 and 12 manifested mixed forms of the disease at necropsy (Table 1).

Altogether, 13 of 46 cats (28.3%) died from FIP between September 2011 and April 2012. During this period, 33 cats (71.7%) appeared to be clinically healthy, and 26 of these asymptomatic cats (78.7%) were positive at least once for FCoV detection in their feces by RT-nPCR. The other seven of these asymptomatic cats were negative for FCoV detection (Table 2).

**Table 2:** Detection and typing of FCoV in fecal samples from healthy cats in the same shelter

**No.**	**FCoV**				**Type**
	**Oct. 2011**	**Feb. 2012**	**Jun. 2012**	**Jul. 2012**	
14	++	++	++	+	untypable
15	-	+	-		untypable
16	++	-	-		untypable
17	++	++	+	++	I
18	++	++	++	++	I
19	-		-	+	untypable
20	-		-	+	untypable
21	-		-	-	
22	++	++	-		untypable
23	+	++	-	+	I
24	-	+	-		untypable
25	++	++	+	+	I
26	-		+	-	untypable
27	++	++	+	+	I
28	++	++	++	+	I
29	-		-	-	
30	-	++	++	-	I
31	-			-	
32	+	++	-	-	I
33	-		++	-	untypable
34	++				I
35	-		+	+	untypable
36	++	++	+	-	I
37			-		
38			+		untypable
39		++	+	+	I
40		-	+	-	untypable
41		+	-	+	untypable
42			+	-	untypable
43			-		
44				-	
45				-	
46			+		untypable

++: FCoV detected in the first round of PCR.

+: FCoV detected only in the nested PCR.

### Type II FIPV were consistently detected in the FIP-succumbed cats

To further investigate the relationship between these seven histopathologically confirmed FIP cats, amplified DNA were typed, sequenced and analyzed. Type II FIPV were detected in all eight of the animals that succumbed to FIP in their swab samples, feces, urine, body effusions, cerebrospinal fluids and tissue homogenates (Table 3). Type II viruses that cause FIP were found not only in the diseased tissue but also in the fecal samples (cat 7, 11, 12 and 13), nasal/oral/conjunctival swab samples (cat 7, 8, 9, 11 and 12), and urine collected by cystocentesis (cat 11) (Table 3). Although there was no necropsy, the ascites from cat 1, the first FIP death in this cat shelter, was available for analysis. This cat was confirmed to be infected with a type II virus. From the healthy animals, only type I or untypable FCoV were detected from the fecal samples (Table 2). Cats 8, 9 and 13 were co-infected with both types of FCoV (Table 3). Although more than one FCoV, i.e., type I, II or untypable viruses, was found to circulate in this multi-cat environment, the eight FIP cats were consistently found to be infected with a type II FCoV, whereas the healthy cats were not (Tables 2 and 3).

**Table 3:** **The characteristics of *3c *genes of FCoV recovered from various specimens of FIP cats**

**Case no.**	**Genotype of FCoV**											***S *gene crossover site**	**Integrity of the *3c *gene**^**b**^						
	***NOC***	***R/F***	***U***	***A/P***	***CSF***	***Li***	***Lu***	***Ki***	***Br***	***Sp***	***Int***		***R/F***	***A/P***	***Li***	***Lu***	***Ki***	***Br***	***Sp***
1				II								4250^a^		intact					
7	II	II		II		II	II	II	II	II		4250	intact		intact	intact	intact		intact
8	II	I				+	II	II	+	-	-								
9	II	I		II		+	II		II	II	+	4250		G210*				G210*	G210*
10						+	+	II	II		II	4250					intact		
11	II	II	II	II	+	II	II	II		+		4250				E47*			
12	II	II		II	II	+	II	II		II	+	4250	G210*	G210*					
13		I/II			+	+	+	II	II	+		4250						Q218*	

*NOC*, nasal/oral/conjunctival swabs; *R/F*, rectal swabs or fecal samples; *A/P*, ascites or pleural effusions; *CSF*, cerebrospinal fluid; *Li*, liver; *Lu*, lung; *Ki*, kidney; *Br*, brain; *Sp*, spleen; *Int*, intestine.

+: FCoV positive but the type of virus cannot be determined.

-: FCoV negative.

^a^: FCoV/NTU2/R/2003; GenBank: DQ160294.

^b^: E47*, G210* and Q218*: truncated 3c proteins with premature stop codons at amino acids 47, 210 and 218 were found, respectively.

### A type II FIPV of the same origin was detected from FIP-succumbed cats

To further investigate the relationship of these disease-associated type II viruses isolated from FIP-succumbed cats, specific primer sets that can specifically amplify from the 3′ end of type II *S* gene to a downstream gene were used to analyze the viral sequences. The identity of the 620 bp amplicons derived from seven type II FIPV was approximately 98.7% to 99.8%. Phylogenetic analysis revealed that the type II FCoV derived from this outbreak were all grouped in a separate cluster, distinct from the other four type II FCoV currently available in the GenBank, i.e., FIPV 79-1146 (GenBank: DQ010921), FCoV 79-1683 (GenBank: JN634064), FCoV DF-2 (GenBank: DQ286389) and FCoV NTU156 (GenBank: GQ152141) (data not shown).

**Figure 1:**
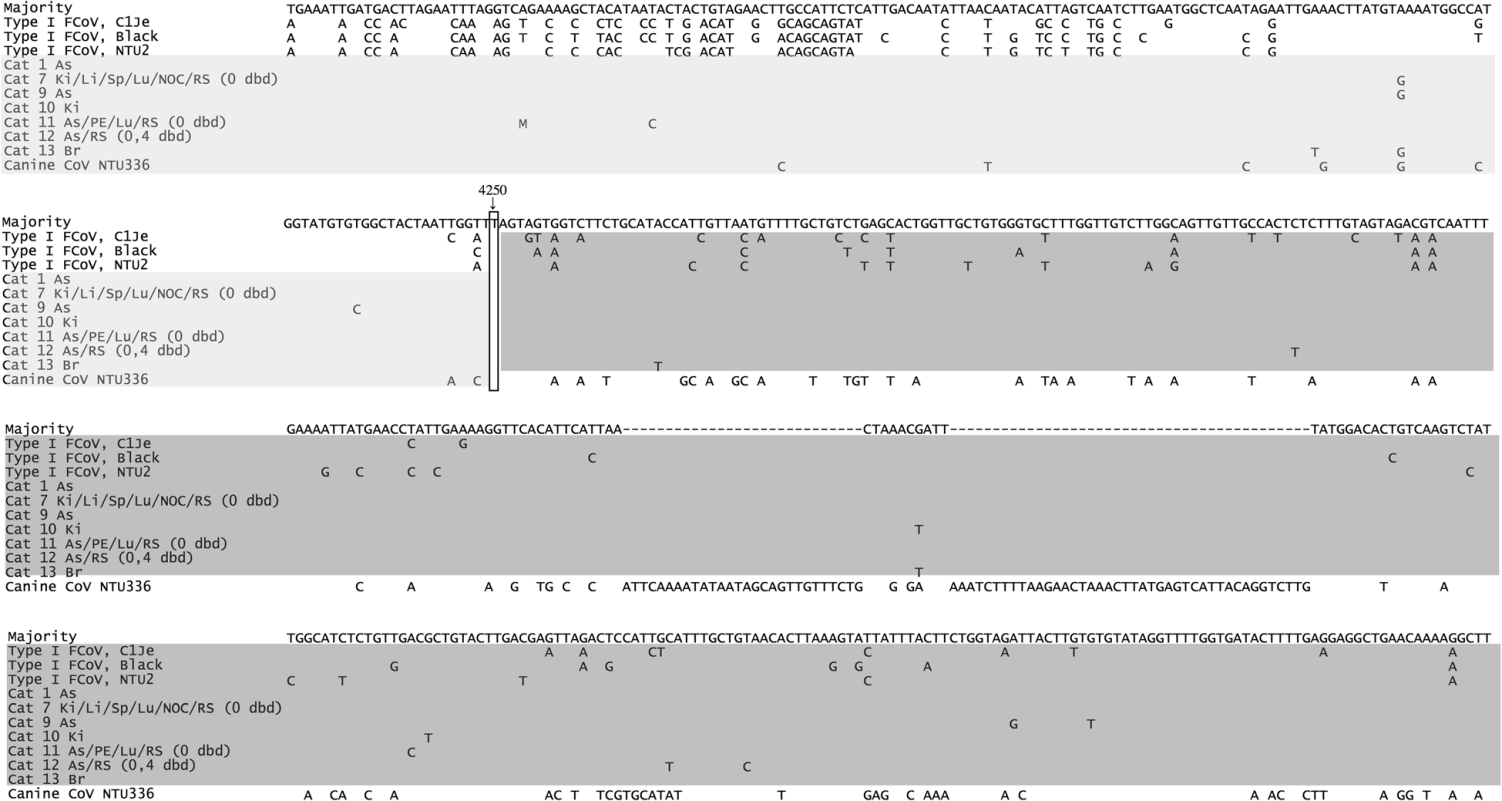
**Recombination of FIPV from cats 1, 7, 9, 10, 11, 12 and 13 on the *S *gene.** The alignment of the 3′ end of the *S* gene to the downstream genes of FCoV isolated from seven FIP cats with type I FCoV and CCoV. The light and dark shaded areas encompass higher similarity to CCoV and type I FCoV, respectively. The putative recombination event occurred at nucleotide 4250 based on the comparison with FCoV NTU2 and is indicated with an arrow. The sequences were obtained from FIPV found in individual samples and tissues and are shown collectively. NOC: nasal/oral/conjunctival swabs; RS: rectal swabs; As: ascites; PE: pleural effusion; Li: liver; Lu: lung; Ki: kidney; Br: brain; Sp: spleen; dbd: days before death. GenBank accession number: FCoV C1Je (GenBank: DQ848678), FCoV Black (GenBank: EU186072), FCoV NTU2 (GenBank: DQ160294) and CCoV NTU336 (GenBank: GQ477367).

A recombination event at the 3′ end of the *S* gene with the putative recombination site located at nucleotide 4250 was identified from all of the type II FCoV derived from the body effusions and tissue homogenate of cats 1, 7, 9, 10, 11, 12 and 13 (Additional file 1) (Table 3). Sequences upstream from this site show a higher similarity to CCoV, whereas sequences downstream from this site were more similar to type I FCoV (Figure 1). These findings highly suggest that the type II FCoV recovered from all of the FIP cats were from a common origin.

### An identical nonsense mutation in *3c* gene was found in two FIP-succumbed cats

**Figure 2:**
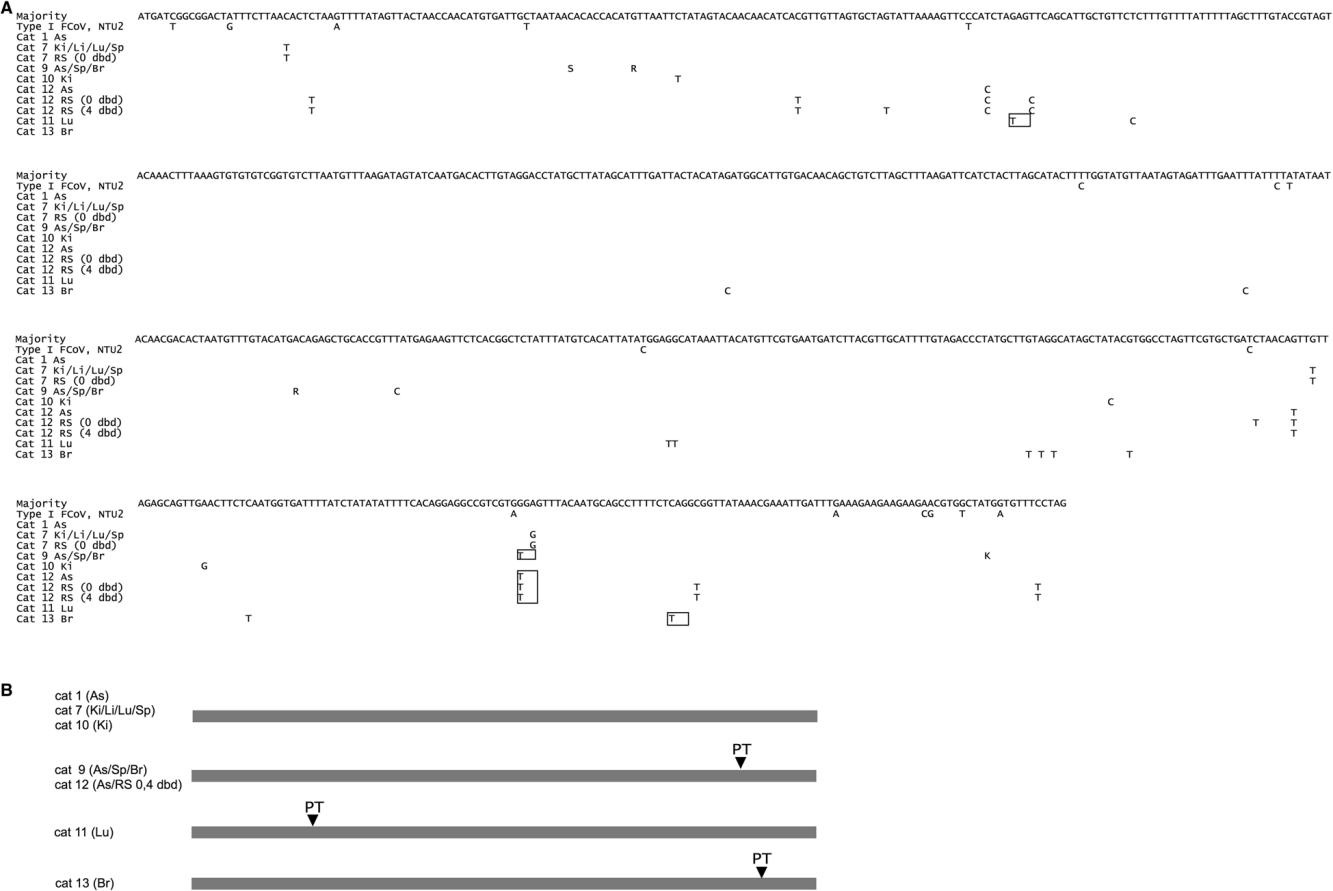
**Alignment of complete *3c *genes of FIPV from cats 1, 7, 9, 10, 11, 12 and 13.** (**A**) Full-length *3c* genes analyzed in this study were aligned with a type I FCoV, FCoV NTU2. The sequences were obtained from FIPV found in individual samples and tissues and are shown collectively. A box represents identified premature stop codons. (**B**) Scheme shows the location of premature stop codons (PT) of *3c* gene of various specimens from different FIP cats.

To further analyze the relationship of these FIPV, *3c* genes, the proposed virulence-associated factor of FIP, were amplified from disease-related type II FCoV. Mutated *3c* genes with an identical premature stop codon at nucleotides 628-630 (amino acid 210, G210*) were found in two FIP cats, including cats 9 (ascites, spleen and brain) and 12 (ascites and rectal swabs from the day it succumbed and four days before) (Figure 2A). It is noteworthy that the FIPV shed from cat 12 harbored an identical nonsense mutation as the virus in its ascites. Intact *3c* genes were detected from cats 1, 7 and 10, which had previously succumbed to FIP. Two more distinct nonsense mutations were identified from cats 11 (E47*) and 13 (Q218*) (Figure 2A-B, Table 3).

### Shedding of the type II FIPV can be detected at the terminal stage of FIP cats

The presence of FCoV was continuously analyzed to elucidate the possible route of shedding and transmission of FIPV. Disease-associated type II FCoV were found to shed through nasal/oral/conjunctival and fecal routes (Table 4). Fecal and nasal/oral/conjunctival shedding of this type II virus can be detected from the 6_th_ day (cat 11) and 4_th_ day (cat 12) respectively, before death. Viremia could be detected during the terminal stage in the cats with FIP, up to 18 days before death, and simultaneous fecal shedding was noted in one cat (cat 12) (Table 4).

**Table 4:** Shedding and serotypes of feline coronavirus detected in FIP cats in the cat shelter

**Cat no.**	**Sample**	**Days before death**																	
		**−80**	**−66**	**−60**	**−57**	**−50**	**−43**	**−36**	**−29**	**−25**	**−23**	**−20**	**−18**	**−14**	**−12**	**−8**	**−6**	**−4**	0^*^
9	Feces		I													I			II
	*NOC* swabs																		II
	Viremia															II			+
	Effusions															II	II		II
11	Feces						-		-					-			II		II
	*NOC* swabs													-			-		II
	Viremia								-					-	-		-		-
	Effusions						+												II
12	Feces	-	+	-		-	-	-	-	-	-	-	-	-	+			II	II
	*NOC* swabs					-	-	-	-	-	-	-	-	-	-			II	II
	Viremia	-		-		-	-	-	-	-	-	-	II	+	+			-	
	Effusions	II																	II

+: FCoV positive; -: FCoV negative.

I, II: Type I or type II FCoV.

^*^: Samples were collected directly prior to euthanasia, except for cat 12, from which the samples were taken after death.

## Discussion

The possibility of horizontal transmission in FIP is generally questioned because (i) the occurrence of FIP is sporadic, and a single cat developing FIP in a multi-cat environment is common [2]; (ii) the internal mutation theory, which describes that FIPV is a mutant generated from enteric FCoV in one cat [12,17]; (iii) there is a lack of evidence of mutant FIPV shedding from FIP cats; and (iv) the mutation of the *3c* gene is unique to each FIP cat [11,13,18]. The current belief is that cats that succumb to FIP do not shed and transmit the FIPV to other cats [11,13,14,18-20]. Our data indicate that this outbreak of FIP was caused by viruses from the same origin. First, all of the FIP losses involved a type II FIPV infection, and the recombination event of these seven type II viruses was mapped to the same site. The recombination sites of the type II viruses currently available in GenBank, i.e., FIPV 79-1146, FCoV 79-1683 and FCoV NTU156, were all unique and occurred independently [9,10]. Second, the FIPV identified in the three kittens that died within the first two months after the onset of fever harbored an intact *3c* gene, whereas the viruses from the cats that survived longer (died four to eight months later) all bore a nonsense mutation, i.e., G210* (cat 9 and 12), E47* (cats 11) and Q218* (cat 13). Because the three nonsense mutations detected in the FIPV from these animals were all located at different sites, the viruses that originally infected these cats should all harbor an intact *3c* gene - like the virus recovered from the kittens that succumbed earlier. After infection, point mutations arose during the replication of the virus in the individual cats, giving rise to FIPV with a *3c* gene bearing nonsense mutations at different locations. The finding that the viruses that were identified not only in the tissues but also in the fecal samples of two cats (cat 9 and 12) harbored an identical mutation in the *3c* gene further confirmed that horizontal transmission had occurred (Table 3). Taken together, all these findings demonstrated that a highly virulent FIPV had spread from animal to animal in a horizontal manner.

This is the first report of a type II FIPV outbreak with evidence of horizontal transmission of a disease-associated FCoV. The outbreak of FIP started after introducing five kittens (cat 1, 3, 4, 8 and 10) into this shelter between June and July of 2011. Because the causative type II viruses with the distinct genetic marker in the S gene were confirmed to be a recombinant of feline and canine coronaviruses and some of the kittens that died earlier were found to have been living closely with dogs between being rescued and entering the shelter, any of these kittens could have been the source of this type II virus. Dogs, and especially young dogs, in shelters often shed large amounts of canine coronavirus in their feces and recombination between cat and dog and dog and pig coronaviruses has been well documented [21-23]. Additionally, these causative type II viruses were detected in many excretions and secretions of cats dying of FIP (Table 3), thus providing a vehicle for cat-to-cat spreading.

Although directly following the first FCoV screening of all the animals in this shelter, cats shedding FCoV were caged separately, and transmission subsequently ceased, the mortality rate for this outbreak was high (28%, 13/46). Three studies regarding outbreak of FIP have been reported previously. In a four-year study conducted in a closed breeding cattery, the average mortality was 17.3% [24], and in a 10-year study of a closed breeding colony, the mortality was 29.4% (5/17) [25]. Another epidemic study conducted in seven catteries/shelters revealed a > 10% mortality rate [20]. The high incidence of FIP in those closed breeding colonies might be influenced by the genetically predisposed breeding stock. In our study, only several FIP cats in this shelter were siblings, and the others were genetically unrelated. Our study demonstrates that without the influence of genetic predisposing factors, the mortality of FIP can still be high in a closed multi-cat environment as long as the spread of the disease-associated FCoV remains undetected.

In this multi-cat environment, three of the FIP cats were infected with not only type II FCoV but also co-infected with type I FCoV (Table 3). Type I FCoV were found only in the fecal samples, whereas type II FCoV were found in the diseased-associated samples, including body effusion, granulomatous tissue homogenates and cerebrospinal fluid. This finding indicates that in these dually infected animals, type II FCoV was the main cause of FIP. This finding is consistent with our previous finding that infection with type II FCoV appears to be significantly correlated with FIP [4].

The presence of FCoV in the whole blood at the terminal stage has been observed previously [26,27]; however, to our knowledge, fecal shedding of FIPV before the end stage of the disease has not been reported prior to this study. Shedding of this type II virus through fecal and nasal/oral/conjunctival routes can be detected in effusive form FIP up to six days before death. Another experimental infection study showed that inoculated viruses could only be recovered for approximately two weeks after inoculation, before clinical signs of disease develop [14]. Taken together, the transmission of FIPV might occur at the onset, before disease manifestation and at the terminal stage. In this outbreak, all the cats were housed together in an open room at the very beginning. After seven cats succumbed sequentially, all FCoV-positive cats were caged individually, and kept separated. The isolation eventually terminated the transmission of the disease. This outbreak, which killed 13 cats, allowed us to determine clearly that FIPV could be transmitted horizontally and showed that isolation of diseased cats should be taken into consideration in a multi-cat environment.

## Competing interests

The authors declare that they have no competing interests.

## Authors’ contributions

YTW performed the sampling and preparation, FCoV detection, typing, *3c* gene amplification and further analysis and prepared the manuscript. BLS supervised the sampling and treatment of all FIP animals and contributed to the preparation of the manuscript. LEH participated in *3c* gene amplification, genetic analysis and the preparation of the manuscript. LLC conceived the study, participated in study design and coordination and contributed to the preparation of the manuscript. All authors read and approved the final manuscript.

## Supplementary Material

Additional file 1 **Recombination site analysis of FIPV from cats 1, 7, 9, 10, 11, 12 and 13 on the *S *gene.** Similarity plot analysis with the Kimura (two-parameter) distance model, neighbor-joining tree model and 100 bootstrap replicates showed a recombination event, and the putative crossover site is indicated with an arrow. Click here for file

## References

[B1] Lai MMC Perlman S Anderson LJ Knipe DM, Howley PM, Griffin DE, Lamb RA, Martin MA, Roizman B, Straus SE Coronaviridae Fields virology 2007 Philadelphia: Lippincott Wiilliams & Wikins 1305 1335

[B2] Pedersen NC A review of feline infectious peritonitis virus infection: 1963-2008 J Feline Med Surg 2009 11 225 258 10.1016/j.jfms.2008.09.008 19254859 PMC7129802

[B3] Pedersen NC Black JW Boyle JF Evermann JF McKeirnan AJ Ott RL Pathogenic differences between various feline coronavirus isolates Adv Exp Med Biol 1984 173 365 380 10.1007/978-1-4615-9373-7_36 6331125

[B4] Lin CN Su BL Wang CH Hsieh MW Chueh TJ Chueh LL Genetic diversity and correlation with feline infectious peritonitis of feline coronavirus type I and II: a 5-year study in Taiwan Vet Microbiol 2009 136 233 239 10.1016/j.vetmic.2008.11.010 19117699 PMC7117496

[B5] Addie DD Schaap IA Nicolson L Jarrett O Persistence and transmission of natural type I feline coronavirus infection J Gen Virol 2003 84 2735 2744 10.1099/vir.0.19129-0 13679608

[B6] Benetka V Kubber-Heiss A Kolodziejek J Nowotny N Hofmann-Parisot M Mostl K Prevalence of feline coronavirus types I and II in cats with histopathologically verified feline infectious peritonitis Vet Microbiol 2004 99 31 42 10.1016/j.vetmic.2003.07.010 15019109 PMC7117137

[B7] Hohdatsu T Okada S Ishizuka Y Yamada H Koyama H The prevalence of types I and II feline coronavirus infections in cats J Vet Med Sci 1992 54 557 562 10.1292/jvms.54.557 1322718

[B8] Kummrow M Meli ML Haessig M Goenczi E Poland A Pedersen NC Hofmann-Lehmann R Lutz H Feline coronavirus serotypes 1 and 2: seroprevalence and association with disease in Switzerland Clin Diagn Lab Immunol 2005 12 1209 1215 16210485 10.1128/CDLI.12.10.1209-1215.2005 PMC1247821

[B9] Lin CN Chang RY Su BL Chueh LL Full genome analysis of a novel type II feline coronavirus NTU156 Virus Genes 2013 46 316 322 10.1007/s11262-012-0864-0 23239278 PMC7089305

[B10] Herrewegh AA Smeenk I Horzinek MC Rottier PJ de Groot RJ Feline coronavirus type II strains 79-1683 and 79-1146 originate from a double recombination between feline coronavirus type I and canine coronavirus J Virol 1998 72 4508 4514 9557750 10.1128/jvi.72.5.4508-4514.1998 PMC109693

[B11] Vennema H Poland A Foley J Pedersen NC Feline infectious peritonitis viruses arise by mutation from endemic feline enteric coronaviruses Virology 1998 243 150 157 10.1006/viro.1998.9045 9527924 PMC7131759

[B12] Rottier PJ Nakamura K Schellen P Volders H Haijema BJ Acquisition of macrophage tropism during the pathogenesis of feline infectious peritonitis is determined by mutations in the feline coronavirus spike protein J Virol 2005 79 14122 14130 10.1128/JVI.79.22.14122-14130.2005 16254347 PMC1280227

[B13] Chang HW de Groot RJ Egberink HF Rottier PJ Feline infectious peritonitis: insights into feline coronavirus pathobiogenesis and epidemiology based on genetic analysis of the viral 3c gene J Gen Virol 2010 91 415 420 10.1099/vir.0.016485-0 19889934

[B14] Stoddart ME Gaskell RM Harbour DA Gaskell CJ Virus shedding and immune responses in cats inoculated with cell culture-adapted feline infectious peritonitis virus Vet Microbiol 1988 16 145 158 10.1016/0378-1135(88)90039-9 2836990 PMC7117427

[B15] Herrewegh AA de Groot RJ Cepica A Egberink HF Horzinek MC Rottier PJ Detection of feline coronavirus RNA in feces, tissues, and body fluids of naturally infected cats by reverse transcriptase PCR J Clin Microbiol 1995 33 684 689 7751377 10.1128/jcm.33.3.684-689.1995 PMC228014

[B16] Chomczynski P Sacchi N Single-step method of RNA isolation by acid guanidinium thiocyanate-phenol-chloroform extraction Anal Biochem 1987 162 156 159 2440339 10.1006/abio.1987.9999

[B17] Poland AM Vennema H Foley JE Pedersen NC Two related strains of feline infectious peritonitis virus isolated from immunocompromised cats infected with a feline enteric coronavirus J Clin Microbiol 1996 34 3180 3184 8940468 10.1128/jcm.34.12.3180-3184.1996 PMC229479

[B18] Pedersen NC Liu H Scarlett J Leutenegger CM Golovko L Kennedy H Kamal FM Feline infectious peritonitis: role of the feline coronavirus 3c gene in intestinal tropism and pathogenicity based upon isolates from resident and adopted shelter cats Virus Res 2012 165 17 28 10.1016/j.virusres.2011.12.020 22280883 PMC7114484

[B19] Foley JE Poland A Carlson J Pedersen NC Patterns of feline coronavirus infection and fecal shedding from cats in multiple-cat environments J Am Vet Med Assoc 1997 210 1307 1312 9143535

[B20] Foley JE Poland A Carlson J Pedersen NC Risk factors for feline infectious peritonitis among cats in multiple-cat environments with endemic feline enteric coronavirus J Am Vet Med Assoc 1997 210 1313 1318 9143536

[B21] Stavisky J Pinchbeck G Gaskell RM Dawson S German AJ Radford AD Cross sectional and longitudinal surveys of canine enteric coronavirus infection in kennelled dogs: a molecular marker for biosecurity Infect Genet Evol 2012 12 1419 1426 10.1016/j.meegid.2012.04.010 22543007 PMC7106024

[B22] Decaro N Mari V Elia G Addie DD Camero M Lucente MS Martella V Buonavoglia C Recombinant canine coronaviruses in dogs, Europe Emerg Infect Dis 2010 16 41 47 10.3201/eid1601.090726 20031041 PMC2874359

[B23] Decaro N Buonavoglia C An update on canine coronaviruses: viral evolution and pathobiology Vet Microbiol 2008 132 221 234 10.1016/j.vetmic.2008.06.007 18635322 PMC7117484

[B24] Potkay S Bacher JD Pitts TW Feline infectious peritonitis in a closed breeding colony Lab Anim Sci 1974 24 279 289 4362875

[B25] Watt NJ MacIntyre NJ McOrist S An extended outbreak of infectious peritonitis in a closed colony of European wildcats (Felis silvestris) J Comp Pathol 1993 108 73 79 10.1016/S0021-9975(08)80229-0 8386199 PMC7130280

[B26] de Groot-Mijnes JD van Dun JM van der Most RG de Groot RJ Natural history of a recurrent feline coronavirus infection and the role of cellular immunity in survival and disease J Virol 2005 79 1036 1044 10.1128/JVI.79.2.1036-1044.2005 15613332 PMC538555

[B27] Tsai HY Chueh LL Lin CN Su BL Clinicopathological findings and disease staging of feline infectious peritonitis: 51 cases from 2003 to 2009 in Taiwan J Feline Med Surg 2011 13 74 80 10.1016/j.jfms.2010.09.014 21216644 PMC7129202

