# Supplementary figures and images for: An outbreak of feline infectious peritonitis in a Taiwanese shelter: epidemiologic and molecular evidence for horizontal transmission of a novel type II feline coronavirus
Source: Vet Res. 2013 Jul 17;44(1):57. doi: 10.1186/1297-9716-44-57 (PMC3720556; doi:10.1186/1297-9716-44-57)

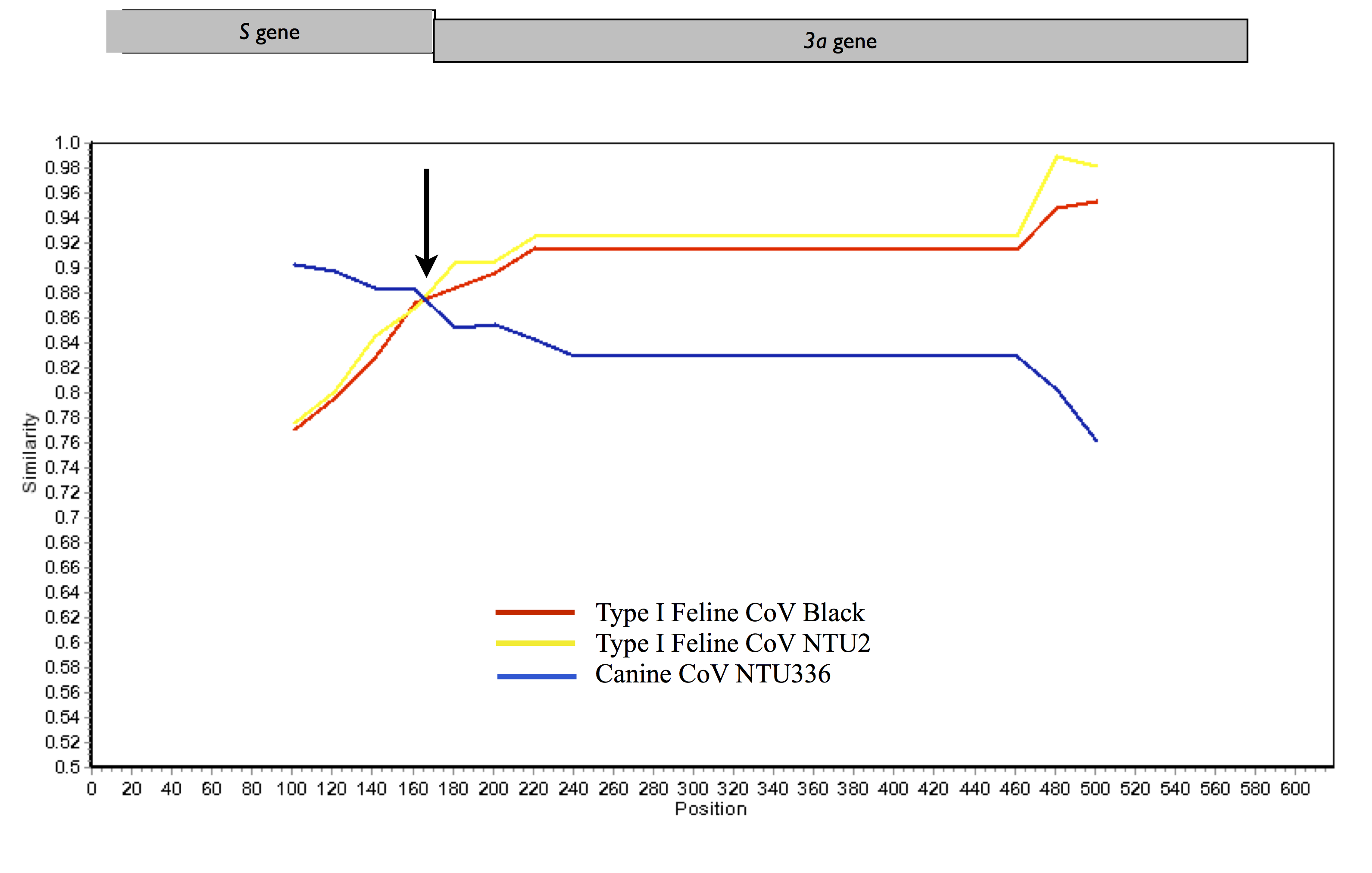

Supplement: Additional file 1 — Recombination site analysis of FIPV from cats 1, 7, 9, 10, 11, 12 and 13 on the S gene. Similarity plot analysis with the Kimura (two-parameter) distance model, neighbor-joining tree model and 100 bootstrap replicates showed a recombination event, and the putative crossover site is indicated with an arrow. [file 1297-9716-44-57-S1.tiff]
